# HOME CARE AND QUALITY OF LIFE AMONG COMMUNITY-DWELLING FRAIL OLDER ADULTS IN ISRAEL: A MULTIPLE MEDIATOR MODEL

**DOI:** 10.1093/geroni/igad104.2602

**Published:** 2023-12-21

**Authors:** Rabia Khalaila

**Affiliations:** Zefat Academic college, Zefat, HaZafon, Israel

## Abstract

The aim of the current study is to examine the association between two types of home care provision (combined formal and informal home care vs. family-only home care) and quality of life (QoL) of older adults, as well as the mediating effects of loneliness, social isolation and satisfaction with family relationships and support. A convenience sample of 360 Israeli adults aged 65+ responded to questionnaires. Using bootstrapping, we tested the strength and significance of the conditional indirect effects of the four simultaneous mediators. The results showed that combined formal and informal home care (vs. family-only home care) for older adults was associated with lower QoL of care-recipients, and was fully mediated by feelings of loneliness, social isolation, and low satisfaction with family support. Satisfaction with family relationships did not emerge as a mediator but was positively associated with QoL. Formal home care might reduce the QoL of care-recipients by increasing their feelings of loneliness, social isolation and reduced perceived family support. Practitioners should encourage family members to continue with family regular support and contact alongside the use of formal home care in order to maintain satisfaction and QoL of older relatives.

